# Diffuse Pulmonary Meningotheliomatosis Mimicking Multiple Lung Metastases of Prostate Cancer: A Case Report

**DOI:** 10.1002/rcr2.70176

**Published:** 2025-04-15

**Authors:** Yuta Sakano, Shotaro Hashimoto, Toyofumi Abe, Kaoru Yamazaki, Saki Kidaka, Satoshi Katsura, Taro Okumura, Kenichi Goto, Shuhei Kosaka, Masato Morimoto, Shigeki Fujita, Michio Shigematsu

**Affiliations:** ^1^ Department of Respirology Sumitomo Hospital Osaka Japan; ^2^ Department of Thoracic Surgery Sumitomo Hospital Osaka Japan; ^3^ Department of Urology Sumitomo Hospital Osaka Japan; ^4^ Department of Pathology Sumitomo Hospital Osaka Japan

**Keywords:** diffuse pulmonary meningotheliomatosis, ground‐glass nodules, metastatic lung cancer, minute pulmonary meningothelial‐like nodule, multiple pulmonary nodules

## Abstract

Diffuse pulmonary meningotheliomatosis (DPM) is a benign pulmonary disease characterised by widespread bilateral minute pulmonary meningothelial‐like nodules that often appear as multiple ground‐glass nodules on chest imaging. We present the case of a 64‐year‐old man with prostate cancer who was found to have multiple pulmonary nodules during cancer staging and was initially suspected to have lung metastases. A surgical lung biopsy confirmed the diagnosis of DPM, allowing the patient to receive curative treatment for localised prostate cancer. DPM is a rare condition reported to be possibly associated with malignancy; however, little is known about its pathogenesis. This case demonstrates the importance of considering DPM in the differential diagnosis of multiple pulmonary nodules, including lung metastases, and the need for further research.

## Graphical abstract

In this report, we experienced a case of diffuse pulmonary meningotheliomatosis (DPM), rare benign multiple pulmonary nodules. This case demonstrates the importance of considering DPM in the differential diagnosis of multiple pulmonary nodules, including metastatic lung cancer.

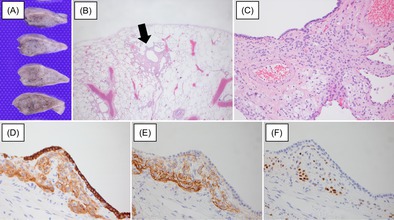

## 1 Introduction

Diffuse pulmonary meningotheliomatosis (DPM) is a benign and rare disease that presents as diffuse pulmonary nodules on imaging. It is characterised by widespread bilateral minute pulmonary meningothelial‐like nodules (MPMNs). A 64‐year‐old man presented with multiple pulmonary nodules during prostate cancer staging and was suspected to have lung metastases. We performed a lung biopsy and diagnosed the lesion as DPM. Therefore, the patient was able to undergo cancer treatment. We believe that DPM should be considered in the differential diagnosis of lung metastases.

## 2 Case Report

**FIGURE 1:**
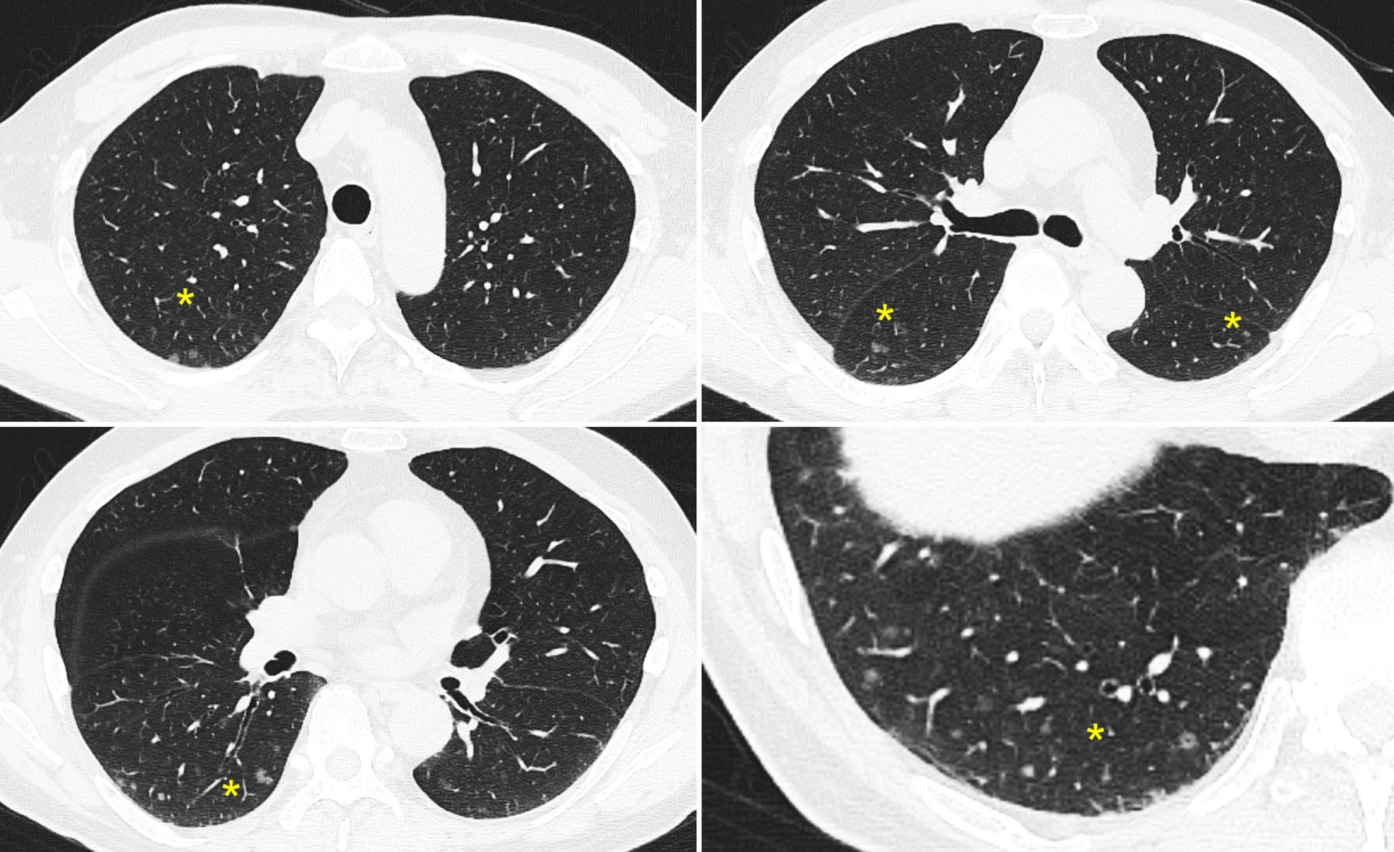
High‐resolution chest computed tomography showed multiple diffuse small ground‐glass nodules at the peripheral zone of each lung (yellow asterisks), some of which demonstrate central cavitation.

A 64‐year‐old man with no history of smoking was referred to our department due to multiple ground‐glass nodules (GGNs) detected by chest computed tomography (CT). He was diagnosed with prostate cancer (cT2a, N0, Mx, Gleason score 8 [4 + 4], adenocarcinoma) and had just initiated neoadjuvant androgen deprivation therapy with bicalutamide and leuprorelin. The patient was scheduled to undergo heavy‐ion radiotherapy as curative treatment. However, a staging CT performed after the start of hormone therapy revealed multiple 1–5 mm GGNs in both lungs with the possibility of lung metastases (Figure 1). There was no evidence of pleural effusion or mediastinal lymphadenopathy. Since heavy‐ion radiotherapy is only indicated for localised prostate cancer, the treatment was postponed. Thus, it was essential to determine whether the GGNs represented lung metastases.

The patient had no respiratory symptoms. In laboratory examination, tumour markers were in the normal range, serum carcinoembryonic antigen (CEA) was 4.4 ng/mL, pro‐gastrin‐releasing peptide (Pro‐GRP) was 49.7 pg/mL, and cytokeratin 19 fragment (CYFRA) level was < 1.0. The hormonal therapy decreased the prostate‐specific antigen (PSA) from 14.11 to 1.19 ng/mL. Inflammatory markers, antinuclear antibody (ANA), rheumatoid factor, anti‐cyclic citrullinated peptide (CCP) and anti‐Sjögren's syndrome type A (SSA) antibody were all negative. Interferon‐gamma release assay (IGRA) for tuberculosis, β‐D‐glucan, and cryptococcal antibody were negative as well. Pulmonary function test showed no abnormalities.

**FIGURE 2:**
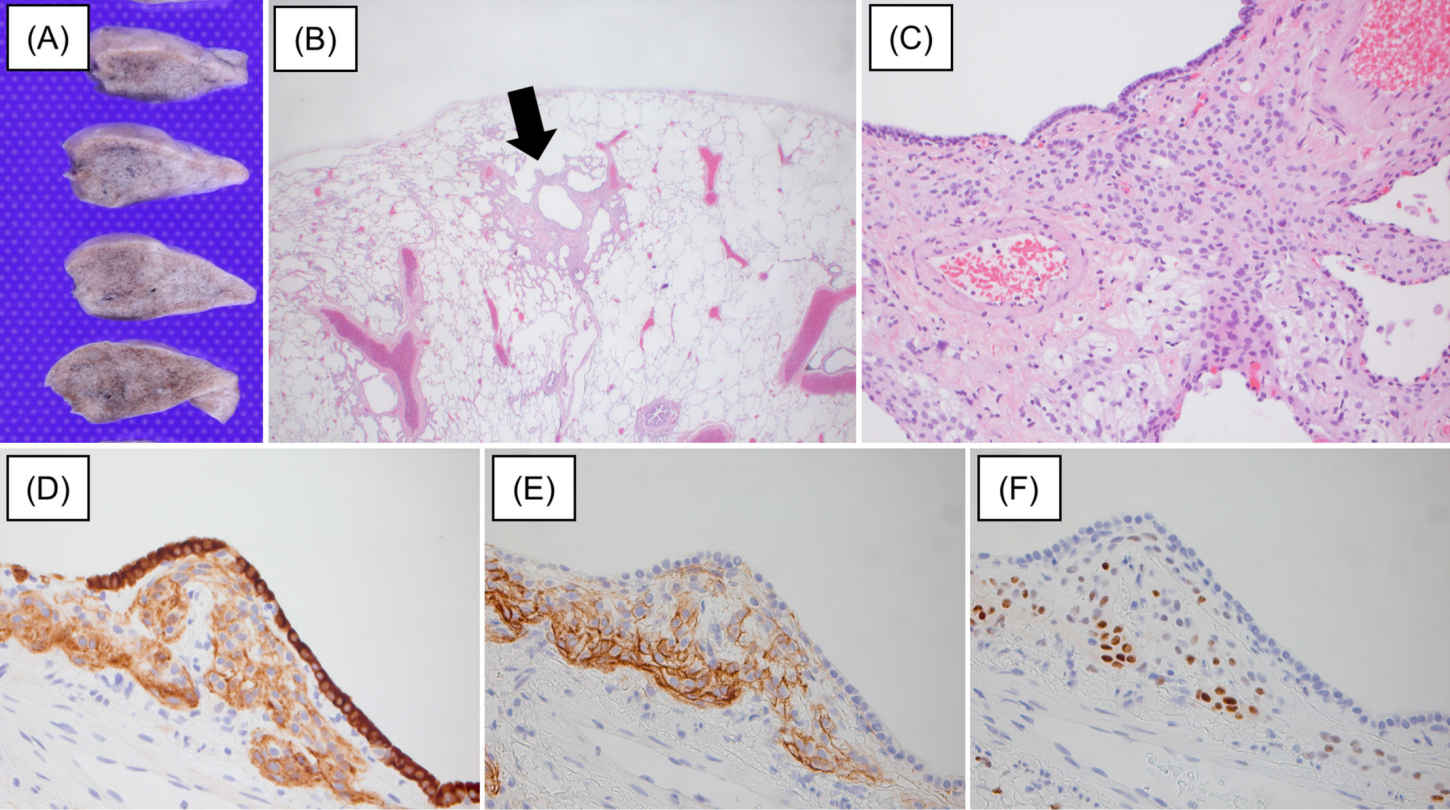
The patient received a lung biopsy by video‐assisted thoracic surgery. The lesion was resected from the edge of the left lower lung, and no macroscopic abnormalities were observed (A). A surgical lung biopsy showed irregular proliferation of meningothelial‐like cells (black arrow) (B), haematoxylin eosin stain magnification ×1.25, oval‐to‐spindle cells with indistinct cellular borders and uniform oval nuclei (C), haematoxylin eosin stain magnification ×20, positive stain for epithelial membrane antigen (EMA) (D), CD56 (E) and progesterone receptors (PgR) (F).

We first performed a transbronchial lung biopsy (TBLB), but no specific findings were observed. In addition, the patient underwent contrast‐enhanced CT (chest to pelvis), contrast‐enhanced brain magnetic resonance imaging (MRI), esophagogastroduodenoscopy (EGD), colonoscopy, and thyroid ultrasonography to detect the presence of adenocarcinoma, but no abnormalities were observed. Therefore, a lung biopsy using video‐assisted thoracic surgery (VATS) was performed to exclude lung metastases from prostate cancer. The lesion was resected from the edge of the lower left lung (Figure 2A). Pathology showed no malignant cells but multiple well‐circumscribed interstitial meningothelial‐like nodules (Figure 2B–D). Immunohistochemistry revealed that the lesion was positive for epithelial membrane antigen (EMA), CD56, progesterone receptors (PgR) and vimentin (Figure 2D–F) and negative for synaptophysin, chromogranin, and NKX3.1. The tissue culture of the resected lung was negative, including mycobacteria and fungi. Based on these findings, we diagnosed the GGNs in this patient as DPM. Thus, his prostate cancer was localised, and he was able to receive localised heavy‐ion radiotherapy. During the one‐year follow‐up, there has been no recurrence of prostate cancer, and the DPM has remained stable.

## 3 Discussion

MPMNs were first reported in 1960 by Korn et al. and were thought to be chemodectomas [1]. However, ultrastructural and immunohistochemical analyses revealed a resemblance to meningothelial cells; therefore, they were named MPMNs [2]. MPMNs are generally benign and detected on chest CT scans performed for other indications [3]. Patients with MPMNs usually have no symptoms and do not develop malignancy [3].

The condition is known as DPM if MPMNs spread diffusely bilaterally in the lungs. DPM is extremely rare; its pathology is not well known, commonly reported in female patients (female‐to‐male ratio of 10:1), and is diagnosed at a mean age of 60 years old (range 30–80 years) [4]. Notably, this case represents a rare instance of DPM occurring in a male patient, whereas most reported cases of DPM are PgR‐positive and are hypothesised to be associated with hormonal imbalances in females [4]. In general, sex hormone imbalances are known to increase the risk of prostate cancer and may have played a role in this case. Although this discussion is based on a single case, further research on the relationship between DPM and sex hormones may be warranted.

The chest CT image of DPM shows multiple nodules randomly distributed predominantly in the peripheral zone of both lungs, some of which have central cavitation, as in this case [3]. In general, GGNs remain unchanged, and a review reported that all 44 patients with DPM were alive and well (range 38–99 months) [4]. A definitive diagnosis of DPM is rarely made using bronchoscopy alone and often requires surgical lung biopsy and immunohistochemical staining of the tissue [4].

A chest CT scan usually detects DPM and is necessary to exclude other diseases presenting with multiple GGNs. In this case, the patient had no respiratory symptoms, and laboratory examinations showed no evidence of infection or autoimmune disease; therefore, these conditions were ruled out. Laboratory examination, EGD, colonoscopy, and thyroid ultrasound were performed to identify lung metastases due to cancers other than prostate cancer; however, no abnormalities were found. Based on these findings, follow‐up was considered; however, a lung biopsy was performed because delaying prostate cancer treatment would be unfavourable for the patient. As a result, we identified these GGNs as a rare benign disease, DPM, and could offer a curative treatment for the cancer.

DPM has been reported to have the possibility of occurring in association with cancer; in a study reporting five cases of DPM, Suster et al. noted that three patients had a history of malignancy [3]. Mizutani et al. reported that MPMNs are more frequently found in patients with malignant lung disease than in those with benign lung disease [5]. Whether DPM appeared before prostate cancer in this case is unknown. Thus, it may be necessary to search for malignancy when a patient is incidentally found to have DPM.

In conclusion, DPM is a rare and benign disease with multiple nodules. However, patients benefit from prompt lung biopsy when investigating potential metastatic lung cancer. Further studies are required to establish an association between DPM and malignancy.

## Author Contributions

Yuta Sakano and Shotaro Hashimoto participated in study design and data interpretation. Yuta Sakano drafted the manuscript. Shotaro Hashimoto, Shuhei Kosaka, and Masato Morimoto performed the surgical lung biopsy. Toyofumi Abe treated prostate cancer. Kaoru Yamazaki and Shigeki Fujita examined the pathologies. All authors critically revised the manuscript, commented on drafts of the manuscript, and approved the final report.

## Ethics Statement

The authors declare that appropriate written informed consent was obtained for the publication of this manuscript and accompanying images.

## Conflicts of Interest

The authors declare no conflicts of interest.

## Data Availability

The data that support the findings of this study are available on request from the corresponding author. The data are not publicly available due to privacy or ethical restrictions.

## References

[1] D. Korn , K. Bensch , A. A. Liebow , and B. Castleman , “Multiple Minute Pulmonary Tumors Resembling Chemodectomas,” American Journal of Pathology 37 (1960): 641–672. 13753180 PMC1942284

[2] M. J. M. D. Gaffey , E. M. D. Stacey , and F. B. M. D. Askin , “Minute Pulmonary Meningothelial‐Like Nodules: Clinicopathologic Study So‐Called Minute Pulmonary Chemodectoma,” American Journal of Surgical Pathology 12, no. 3 (1988): 167–175. 2830799 10.1097/00000478-198803000-00001

[3] S. Suster and C. A. Moran , “Moran CA: Diffuse Pulmonary Meningotheliomatosis,” American Journal of Surgical Pathology 31, no. 4 (2007): 624–631. 17414111 10.1097/01.pas.0000213385.25042.cf

[4] L. Melocchi , G. Rossi , M. Valli , et al., “Diffuse Pulmonary Meningotheliomatosis: Clinic‐Pathologic Entity or Indolent Metastasis From Meningioma (Or Both)?,” Diagnostics (Basel) 13, no. 4 (2023): 802. 36832290 10.3390/diagnostics13040802 PMC9955492

[5] E. Mizutani , K. Tsuta , A. M. Maeshima , H. Asamura , and Y. Matsuno , “Minute Pulmonary Meningothelial‐Like Nodules: Clinicopathologic Analysis of 121 Patients,” Human Pathology 40, no. 5 (2009): 678–682. 19144385 10.1016/j.humpath.2008.08.018

